# Chemogenomics driven discovery of endogenous polyketide anti-infective compounds from endosymbiotic *Emericella variecolor* CLB38 and their RNA secondary structure analysis

**DOI:** 10.1371/journal.pone.0172848

**Published:** 2017-02-28

**Authors:** H. C. Yashavantha Rao, Devaraju Rakshith, Ballagere Puttaraju Harini, Doddahosuru Mahadevappa Gurudatt, Sreedharamurthy Satish

**Affiliations:** 1 Microbial Drugs Laboratory, Department of Studies in Microbiology, University of Mysore, Manasagangotri, Mysore, Karnataka, India; 2 Department of Zoology, Bangalore University, Jnana Bharathi Campus, Bangalore, Karnataka, India; 3 Department of Studies in Organic Chemistry, University of Mysore, Manasagangotri, Mysore, Karnataka, India; Tallinn University of Technology, ESTONIA

## Abstract

In the postgenomic era, a new strategy for chemical dereplication of polyketide anti-infective drugs requires novel genomics and chromatographic strategies. An endosymbiotic fungal strain CLB38 was isolated from the root tissue of *Combretum latifolium* Blume (Combretaceae) which was collected from the Western Ghats of India. The isolate CLB38 was then identified as *Emericella variecolor* by its characteristic stellate ascospores culture morphology and molecular analysis of ITS nuclear rDNA and intervening 5.8S rRNA gene sequence. ITS2 RNA secondary structure modeling clearly distinguished fungal endosymbiont *E*. *variecolor* CLB38 with other lifestyles in the same monophyletic clade. Ethyl acetate fraction of CLB38 explored a broad spectrum of antimicrobial activity against multidrug resistant pathogens. Biosynthetic PKS type-I gene and chromatographic approach afford two polyketide antimicrobial compounds which identified as evariquinone and isoindolones derivative emerimidine A. MIC of purified compounds against test microorganisms ranged between 3.12 μg/ml and 12.5 μg/ml. This research highlights the utility of *E*. *variecolor* CLB38 as an anticipate source for anti-infective polyketide metabolites evariquinone and emerimidine A to combat multidrug resistant microorganisms. Here we demonstrates a chemogenomics strategy via the feasibility of PKS type-I gene and chromatographic approach as a proficient method for the rapid prediction and discovery of new polyketides compounds from fungal endosymbionts.

## Introduction

Novel antimicrobial drugs derived from fungal endosymbionts with the unique and targeted mode of action are fundamental to fight against multidrug-resistant microorganisms[1].Despite the current focus on synthetic chemicals, natural products assist as a continuing source in search of new antimicrobial drugs, retaining an immense impact on modern medicine [2]. Endosymbiotic fungi are an eclectic group of microbes having the power to chemically colligate the bridge between microbes and medicinal plants due to their relatively high metabolic versatility [3].They securely establish endophyte–endophyte and plant-endophyte interactions, which play a vital role in the biosynthesis of anti-infective metabolites [4]. They are highly considered as unexploited drug sources capable of producing novel anti-infective metabolites [5].

Biodiscovery of natural drugs and development are resource and time consuming processes [6]. Application of chemogenomics strategy may provide selective information to predict the nature of antimicrobial compounds during the bioprospecting of endosymbiotic fungi for novel metabolites. Furthermore, microbial genome mining revealed the presence of numerous secondary metabolites gene clusters, displaying a discrepancy between the numbers of putative genes involved in secondary metabolism [7,8]. Advanced approaches in searching for rapid identification of polyketide metabolites from fungal endosymbionts require novel genomics and chemical investigation. Polyketides constitute a diverse group of secondary metabolites found across bacteria, fungi, plants and some marine microorganisms which play an important role in drug discovery from natural resources [9]. They governed by multi-domain enzymes which catalyze iterative events to frame a polyketide molecule [10]. Several antimicrobial drugs in the market are of polyketide origin including antibiotics tetracycline and erythromycin, anticholesterol drug lovastatin, anticancer drug epothilone B and immunosuppressant rapamycin [11].

The genus *Emericella* was first described by Berkeley [12] (1857) with *Emericella variecolor* (anamorph: *Aspergillus stellatus* syn. *A*. *variecolor*) [13]. *Emericella* is a genus containing species of considerable interest because of its well elucidated genetics of *E*. *nidulans* [14] and due to some species which reported to produce penicillin [15]. Member of the genus *Emericella* is an ecologically versatile and industrially important group of fungi. It is well known to produce diverse bioactive compounds like cytotoxic sesterterpenes [16] stromemycin [17], asperthecin, shamixanthones, sterigmatocystin, andebenin A, B, C as well as xanthones with antimicrobial, immune stimulant and calmodulin inhibition activities to mention in few [18,19,20]. In the present research, *Combretum latifolium* Blume was selected for the isolation of *E*. *variecolor*.

*C*. *latifolium* Blume is a climbing shrub known as Man daeng or Uat chueak which has great medicinal values [21]. Stem and bark of this shrub were used as insecticides [22], where as leaf juice is used to cure dysentery, pneumonia and goitar [23]. The major components present in the volatile oil of *C*. *latifolium* Blume were hexahydrofarnesyl acetone, isophytol, palmitic acid, neophytadiene and n-nonacosane [24]. Therefore, the present study was carried out to employ genomic and metabolomic strategy as a rapid screening mini tool for detecting PKS genes and its antimicrobial metabolites to combat multi-drug resistant pathogens. Here we discuss the merits of this genomic and metabolomic approach for the rapid detection of PKS-type I genes which facilities biodiscovery of polyketide antimicrobial compounds.

## Materials and methods

### Study site characteristics and selection of plant

*C*. *latifolium* Blume was collected from Pushpagiri Sanctuary (12°35′N 75°40′E, elevation 1748 m) located in the Western Ghats of Coorg, Karnataka which is covered with evergreen forests and shoal grassland habitat. The imposing Kumaraparvata peak forms the core of this Sanctuary with dense evergreen forests. The Western Ghats are the mountain ranges that runs almost parallel to the Western coast of Indian peninsula located entirely in India. It is considered as one of the eight ‘hottest hotspots’ of biological diversity exits in the world [25]. No specific permissions were required for this location for plant sample collection and the field studies did not involve any endangered or protected species.

### Collection of samples

Healthy and asymptomatic leaf, stem and root tissue samples of *C*. *latifolium* Blume was carefully collected. In order to secure the endosymbiotic nature of the isolate, blunt ends of the stem and root tissues were sealed with wax. All tissue samples were carried in an icebox and stored at 4°C. All tissue samples were used for the isolation of fungal endosymbionts within 24 h of collection.

### Isolation of endosymbiotic fungi

Isolation of endosymbiotic fungi and its secondary metabolites were carried out as described in detail from our previous studies with some modifications [26]. Surface sterilization: Each sample tissue was washed under running tap water for 15 min and dried at room temperature. All samples were washed with distilled water before processing and slight visibly damaged material was discarded. To remove the epiphytic microorganisms, sample tissues were rinsed with 70% ethanol for 2 min, surface sterilized by sodium hypochlorite (4%) for 5 min and again rinsed with 70% ethanol for 30 s. Sample tissues were then washed with sterile double distilled water and kept for surface drying in sterile condition. The tissue samples were cut into small segments (1 cm) and placed on water agar plates (distilled water, 1.5% agar) amended with chloramphenicol (250 ppm), then incubated at 28±2°C for 7 days to till growth initiated [2]. To confirm the success of surface disinfection process, aliquots of the sterile double distilled water from the final rinse were inoculated on the isolation medium plates. The hyphal tips which emerged from the sample tissues were picked and maintained on PDA plates for further studies.

### Fermentation and extraction of antimicrobial metabolite

*E*. *variecolor* CLB38 was cultured in 1 L Erlenmeyer flask containing 250 ml of potato dextrose broth (PDB). The culture was incubated for 30 days at 28°C under static conditions. Culture broth was then filtered to separate mycelium and culture broth. The filtered culture broth was blended thoroughly and centrifuged at 4000 r/min for 10 min in order to obtain pure culture broth. The liquid supernatant was then extracted three times with an equal volume of ethyl acetate and evaporated to dryness under reduced pressure at 45°C using rotary flash evaporator [27].

### Antimicrobial activity

Determination of antimicrobial susceptibility test was carried out by disc diffusion method. Sterile discs (6 mm) impregnated with 20 μl (100 μg/disc) of ethyl acetate extract obtained from the culture broth of *E*. *variecolor* CLB38 were dried in laminar air flow and placed on the surface of the medium which seeded with test human pathogens in Petri plates. A disc as negative control with only 20 μl of ethyl acetate was also placed for each test pathogen and Gentamicin as positive control. The plates were then incubated at 37±2°C and 28±2°C (for test bacteria and fungi respectively). The diameter of the zone of inhibition was recorded [28].

### Statistical analyses

Statistical analysis of results was performed using IBM SPSS version 20. Analysis of variance (one way ANOVA) at value p <0.001 followed by Tukey’s Post Hoc test with p<0.05 was used to determine the significant difference between the results obtained in each experiment.

### Isolation of genomic DNA, PCR amplification and DNA sequencing

*E*. *variecolor*CLB38was cultured in potato dextrose broth for 7 days at 30°C and the mycelium was harvested by vacuum filtration. The chilled mycelia were ground with a pestle and mortar under liquid nitrogen. Then, the grinded mycelia were transferred into a micro centrifuge tube with 1 ml of 2×CTAB extraction buffer and incubated at 65°C for 30 min with gentle swirling. After centrifugation at 10000 rpm for 10 min, aqueous phase of the mixture containing total DNA was extracted with an equal volume phenol:chloroform:isoamyl alcohol (25:38:1). Residual phenol was removed by the addition of chloroform:isoamyl alcohol (38:1) twice. Two volume ethanol and 0.1 volume 3 M sodium acetate were added to the aqueous phase of DNA to precipitate and incubated at -20°C over-night. The DNA pellet was then washed with 70% ethanol twice and suspended in 15 μl of TE buffer [29]. PCR amplification was carried according to the protocol of Bhagat et al. (2012) [30] using ITS1 (5′ TCCGTAGGTGAACCTGCGG 3′) and ITS4 (5′ TCCTCCGCTTATTGATATGC 3′) set of universal primers [31]. DNA sequencing was performed using an ABI 3730 sequencer (Applied Biosystems, United States).

### Phylogenetic affiliation

Internal transcribed spacer (ITS) sequence data from strain *E*. *variecolor* CLB38was annotated using Geneious 6.1.6 (2013), Biomatters, Auckland, New Zealand) software and submitted to National Centre for Biotechnology Information(NCBI) GenBank. ITS rDNA sequences with maximum identity to that of strain CLB38were retrieved from NCBI nucleotide database using Basic Local Alignment Search Tool (BLAST) search. ITS sequences were filter-searched and closest resembles sequences were retrieved for phylogenetic analysis. Multiple sequence alignments were performed using CLUSTALW software utilizing default settings and dendrogram was generated by MEGA 4.0 with a bootstrap consensus of 1000 replicates [32].

### RNA secondary structure analysis

The ITS2 sequences of strain CLB38 and its closest matches in the phylogenetic clade were selected to predict the ITS2 RNA secondary structure using mfold server with a preset temperature of 37°C and following conditions: ionic conditions, maximum asymmetry of bulge loop at 30; 1 M NaCl with no divalent ions; maximum number of nucleotides in a bulge or loop limited to 30; percentage sub-optimality number 5; upper bound on number of computed folding 50 and the structure selected from dissimilar output files are with the high negative free energy if several similar structures obtained [26,33].

### Occurrence of biosynthetic polyketide synthase (PKS) genes

Biosynthetic gene clusters encoding PKS keto synthase domain was detected using three sets of degenerate primers: LC1 and LC2c, LC3 and LC5c [34], KS3 and KS4c [35], which are ketosynthase domain degenerate primers were used to amplify the PKS genes of *E*. *variecolor*CLB38. PCR reactions (50 μl) contained 4 μl DNA template, 5 μl 10×PCR buffer, 4 μl 2.5 mM of each dNTPs, 3 μl of each primer, 1 μl of 5 U/μl r*Taq* DNA polymerase and 30 μl deionized water. Thermal cycling program: 5 min at 94°C; 34 cycles of 1 min at 94°C, 1.5 min at 55°C, 3 min at 72°C and 10 min at 72°C.

### Structural and functional analyses of PKS gene

The PKS gene secondary functional and structure prediction was carried out using Iterative Threading Assembly Refinement (I-TASSER) online bioinformatics software [36]. This algorithm modeled based on LOMETS multiple-threading alignment and TASSER iterative simulation [37]. The translated protein sequence was derived from PKS nucleotide sequence using ORF finder and searched for related proteins using BLASTP algorithm [38]. PKS enzyme active sites and its sequence were detected using Swiss-pdb viewer and online-web server Scan prosite tool. Quality assessment of the predicted protein 3D model was estimated using based on the Z-Score and RMSD values. Predicted protein 3D model quality was assessed using Q mean score [39]. The C-score (confidence score), TM-score (template modeling score) and ligand binding sites were determined for the binding bioactive molecule [40].

### Thin layer chromatography–bioautography

Antimicrobial activity of secondary metabolites fromstrainCLB38 was determined by analytical thin-layer chromatography (TLC) bioautography method [41]. Ten microlitres of ethyl acetate fraction of *E*. *variecolor*CLB38was spotted on TLC silica gel plates (TLC, Alugram SIL G/UV_254_; Machereye-Nagel, Duren, Germany) in an optimized solvent system of petroleum ether/ethyl acetate (1:2). The developed TLC sheets were then observed under ultraviolet (UV) light (254 nm). TLC plates were then air dried in sterile condition for the complete removal of solvent traces and ultraviolet radiation sterilized for 30 min. Later, the developed TLC plates were cased in sterile Petri plates overlaid with Brain heart infusion medium containing 0.65% soft agar incorporated with 1 mgml^-1^ concentration of 2,3,5-triphenyl tetrazolium chloride (TTC; Sigma-Aldrich) inoculated with 1% standardized microbial inocula. After 2 h of diffusion at 8°C, Petri plates were incubated for 38 h at 37°C. The zone of inhibition on the active spot was recorded.

### Purification of antimicrobial compounds

Antimicrobial compounds from ethyl acetate extract of CLB38 were purified inthe two-step purification process. First, the concentrated extract was fractionated using silica gel (60–200 mesh; HiMedia, Mumbai, India) column chromatography (50 cm×1 cm) eluted successively with hexane/ethyl acetate(v/v, 100:0, 95:5, 90:10, 80:20, 70:30, 60:40, 50:50, 40:60, 30:70, 20:80, 10:90, 5:95 and 0:100, respectively) to afford 13(F1~F13) eluted fractions. In order to pool similar eluted bioactive fractions, 10 μl of eluted fractions were spotted on TLC silica gel plates separately and observed for similar band pattern under UV light (254 nm). Fractions F3~F5and F8~F10, these two groups of fractions showed similar band patterns differ from each group and exhibited strong antimicrobial activity during TLC bioautography. Therefore, active fractions F3~F5 (Group A) and F8~F10 (Group B) were separately pooled together and re-chromatographed over silica gel column (60–200 mesh; Hi Media, Mumbai, India) eluting in mobile phase methanol/ethyl acetate in order to obtain high purity of the compounds (*v/v*, 20:80, 40:60, 60:40 and 80:20, respectively). From the secondary step of purification, group A and group B eluted fractions exhibited strong antimicrobial activity against MRSA, therefore these active fractions were combined separately and evaporated to dryness.

### Structure elucidation of antimicrobial compounds

Electrospray ionization time-of-flight mass spectrometry (ESI-TOF-MS) was carried out using an LCT liquid chromatogram–mass spectrometer (Micromass Q-Tof). Optical rotation was measured on a JASCO P-1020 polarimeter. NMR spectral analyses were recorded in BRUKER-AV400 spectrometer using CDCl_3_ solvent.

### Minimum inhibitory concentration (MIC) of antimicrobial compounds

MIC of the purified compounds was determined by micro broth dilution assay in 96-well plate method[42]. The purified compound was tested in two-fold dilution of concentration range 200–0.3906 μg/ml. 3-(4,5-dimethylthiazol-2-yl)-2,5-diphenyltetrazolium bromide (MTT; Sigma-Aldrich, Bengaluru, India) and 2,3,5-Triphenyltetrazolium chloride (TTC) were added to each well for test fungi and bacteria respectively, as microbial growth indicators. Gentamicin and nystatin were used as positive controls, whereas medium broth alone served as negative control/sterility control. The lowest concentration of the purified compound with no visible growth as indicated by the growth indicators was determined as MIC.

## Results and discussion

### Isolation of endosymbiotic fungi

**Fig 1:**
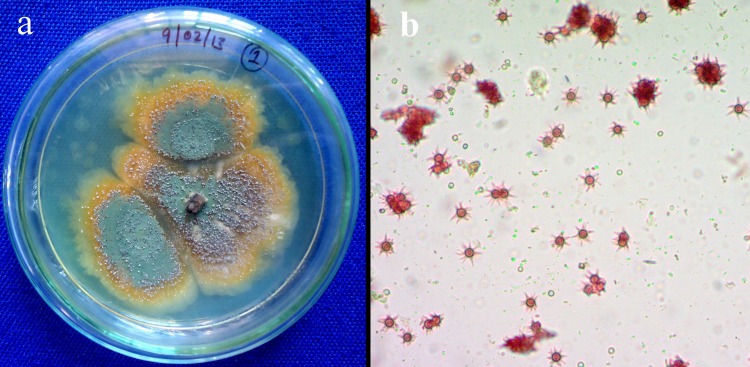
**(a)** Colony morphology of endosymbiotic *Emericella variecolor* CLB38 on potato dextrose agar, **(b)** microscopic stellate ascospores at 40X magnification.

Fungal endosymbionts with the unique and targeted mode of action can impact plant biology through the biosynthesis of diverse chemical entities and modulating gene expression of the host [43]. In the present study, a fungal endosymbiont *E*. *variecolor*CLB38was isolated from the asymptomatic root of *C*. *latifolium* Blume. Growth of bacterial endosymbionts was effectively inhibited by amending the medium with chloramphenicol (250 ppm). In addition, isolation medium plates spread with a final rinse of water do not exhibit any microbial growth even after 10 days of incubation at 28°C. This suggests that the surface sterilization procedure was effective in killing all epiphytic microbes. Thus, the subsequent isolate can be considered as true endosymbiotic fungus [44]. Colony morphology on potato dextrose agar after 7 days of incubation at 28°C initially light green to yellow, later turns to yellow around the corner, margin regular with smooth surface, produced stellate ascospores. The isolated endosymbiotic fungus was then identified as *Emericella* sp. on the basis of morphological and microscopic characteristics (Fig 1). However, molecular analysis of ITS region of rDNA and intervening 5.8S rRNA gene sequence analysis was carried out in order to identify the strain at species level.

### Phylogenetic affiliation

**Fig 2:**
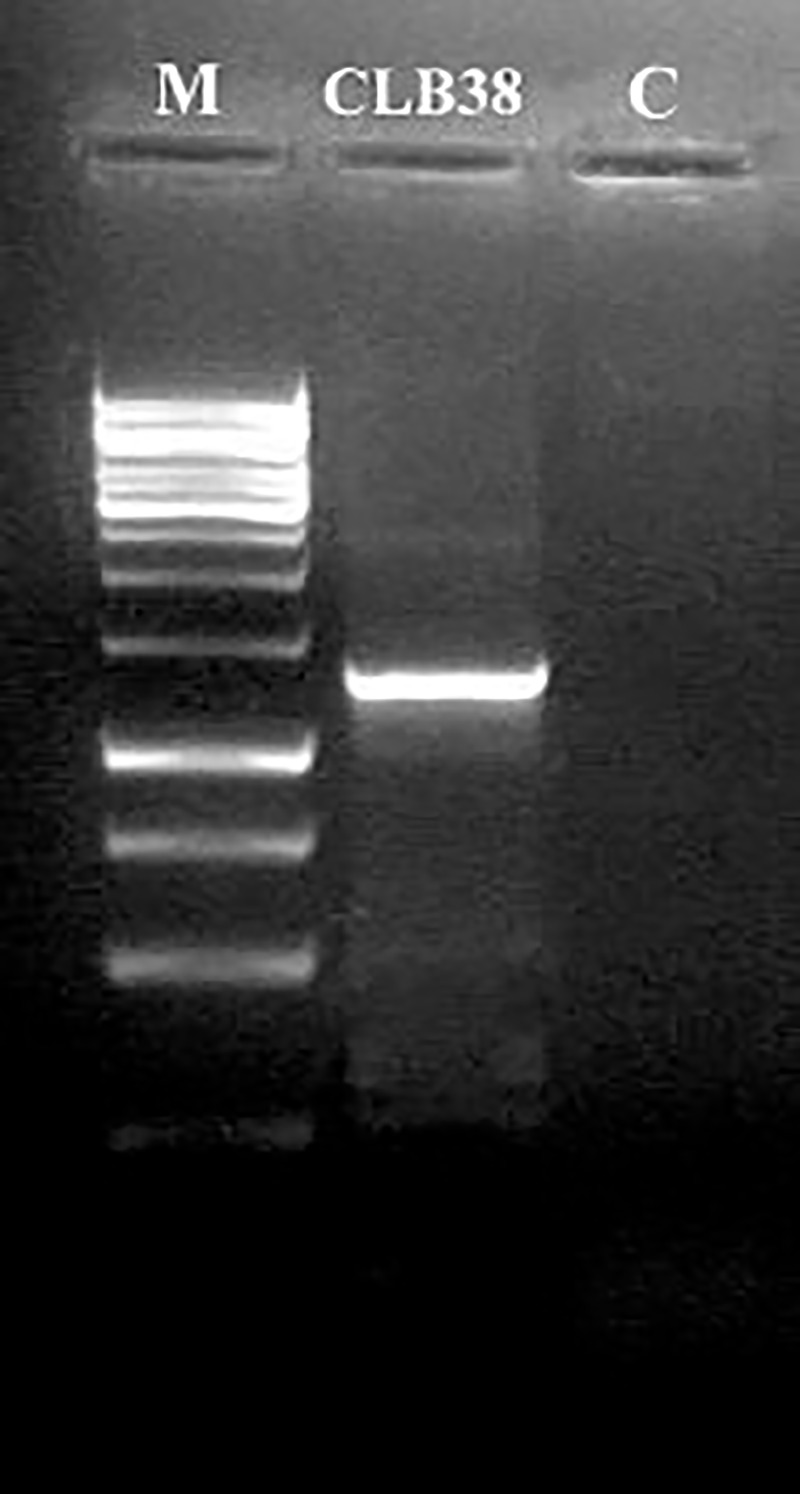
PCR amplification of rDNA from *Emericella variecolor* CLB38 using ITS1 and ITS4 universal primers. Lane: M—100 bp DNA ladder; CLB38 –~575 bp amplicon representing ITS region of rDNA; C–control.

**Fig 3:**
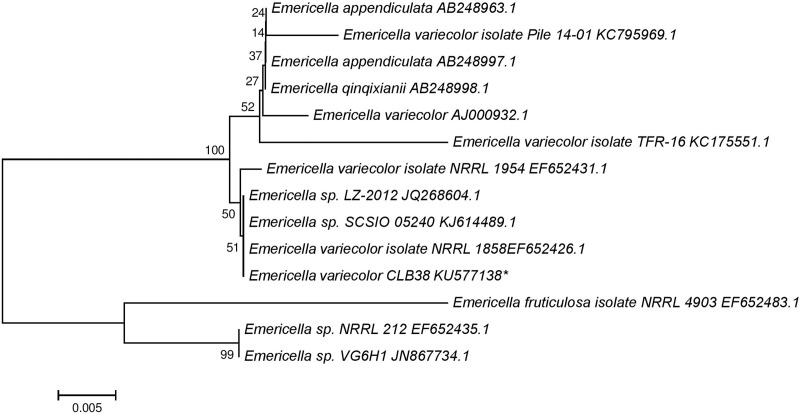
Phylogenetic tree derived from NJ analysis showing the evolutionary relationship of *Emericella variecolor* CLB38 with its closest BLAST hits. Bootstrap values (1000 replications) based on multiple sequence alignment using the MEGA-5 software. Asterisk indicates the isolate obtained in this study.

Molecular identification of the isolate CLB38 was carried out by ITS region of rDNA and intervening 5.8S rRNA gene sequence analysis (Fig 2). PCR amplified ITS region of rDNA was sequenced and aligned with different *Emericella* spp. retrieved from NCBI using BLAST search. ITS sequence alignment retrieved from NCBI database showed several closely resembled sequences. Corresponding neighbor joining (NJ) tree depict clearly that, strain CLB38 fell into the group of *Emericella variecolor* with strong support (Fig 3). BLAST hits and NJ dendrogram generated indicating that isolate CLB38 was more homologous to *Emericella variecolor* isolate NRRL 1858 (GenBank accession no. EF653826.1). There are several previously phylogenetically unstudied endosymbiotic fungi exits [45]; however, morphological identification of fungi has long been usage which is beyond doubt and great significance [46]. ITS rDNA and intervening 5.8S rRNA gene sequence profiling is a cost-effective tool which enables to identify the complex microbial plethora at the species level in exceptional depth [47]. Finally, combined with morphological and phylogenetic data, strain CLB38 was defined as *Emericella variecolor*. The ITS sequence data of this fungus is deposited in GenBank under the accession no. KU577138.

### Occurrence of biosynthetic PKS genes

In addition, the presence of biosynthetic PKS genes encoding keto synthase (KS) domain of strain CLB38 was detected using LC1-LC2c, LC3-LC5c and KS3-KS4c degenerate primers which are specifically for non-reduced, partially reduced and highly reduced KS domains of endosymbiotic fungal polyketide compounds. Genomic DNA of *E*. *variecolor* CLB38 amplified with LC3-LC5c pairs of degenerate primers. Endosymbiotic fungi have been screened genetically to detect the presence of PKS genes as indicators of bioactivity. This encodes an uncharacterized functional enzyme which might suggest a new function for antimicrobial polyketide production [48].The PKS gene sequence data of this fungus is deposited in GenBank under the accession no. KU577139. Lin et al. (2010) [49] reported some fungal endophytes amplified with LC1-LC2c pair of degenerate primers. Endophytic fungal type I polyketides compounds are biosynthesized by multi-domain enzymes, which employ an iterative strategy to build polyketide molecules [10].

### Structural and functional analyses of PKS gene

**Fig 4:**
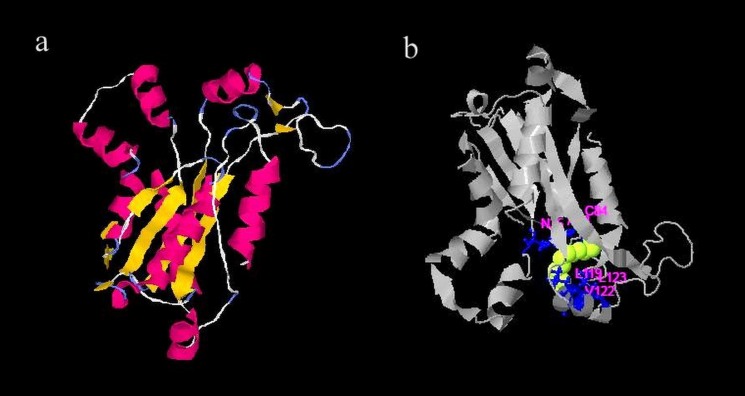
**(a)** 3D modeled structure based on the deduced amino acid sequence of *E*. *variecolor* CLB38 PKS gene using I-TASSER model, **(b)** Predicted ligand-binding site of *E*. *variecolor* CLB38 PKS protein.

Functional annotation and comparative 3D structure modeling and tertiary structure analysis of ketosynthase domain of PKS gene were generated ‘in silico’ (Fig 4).The predicted structure of the PKS protein sequence revealed its primary structure oligo state as homo-dimer. The model resulted conformational stability and likelihood to the template, qualitative values for structural assessment in terms C-score 6.21, TM–score 0.94±0.05 and RMSD score 2.3±1.8Å. It assists pre-selection or endosymbiotic microbial strain prioritization capable of producing new antimicrobial metabolites. ProFunc and active site prediction tools were used to predict the function of modeled protein. I-TASSER server results predict accurate structure and function base on state-of-the-art algorithm [36]. This facilitates PKS gene screening approach as the best method to screen and ascertain the genetic biosynthetic potential for fungal endophytes from the perspective of natural product discovery and might serve as indicators of bioactivities [34,49].

### RNA secondary structure analysis

**Fig 5:**
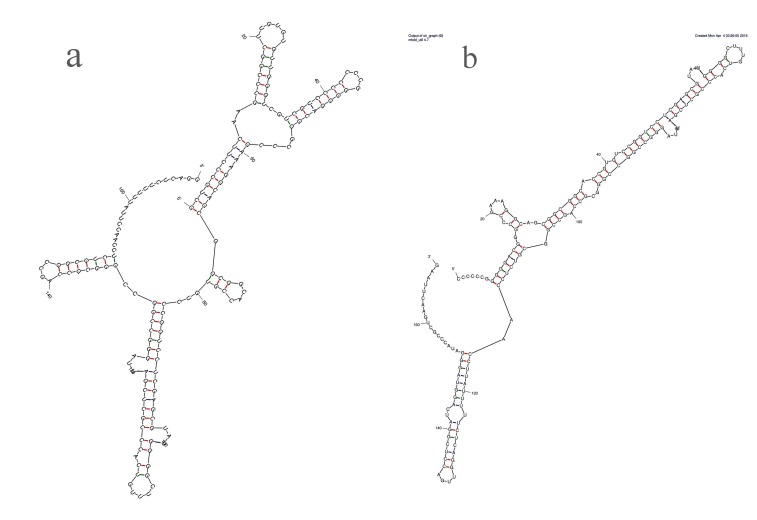
**ITS2 RNA secondary structure of (a) *Emericella variecolor* isolate NRRL 1858 and (b) *Emericella variecolor* CLB38**.

ITS2 RNA secondary structure prediction of *E*. *variecolor* CLB38 with its close phylogeny clade member of *E*. *variecolor* NRRL 1858 (EF652426) was generated in silico. The ITS2 sequence was used as markers in molecular systematics and phylogenetic reconstruction [26]. The generated RNA secondary structures showed structural differences between these two strains.ITS2 RNA secondary structure of *E*. *variecolor* NRRL 1858 displays branched unpaired region where as isolate CLB38 appeared linear in structure and also variations in stem/loop transition were observed (Fig 5). ITS2 RNA structures are sensible to single base changes which in turn can affect hydrogen base pairing in stem and loop secondary structures [50].A non-conserved mismatches might altered base-pairing and stem to loop transitions by counterparts [42,51]. Studies have reported many fungi might express different life-styles in response to environmental factors or host genotype [52]. These findings might suggest that *E*. *variecolor* CLB38 differ by dangling region and unpaired variation of *E*. *variecolor* NRRL 1858 due to their endosymbiotic nature. Therefore, CLB38 is a unique potential source of novel antimicrobial drugs to combat multi drug resistant pathogens.

### Antimicrobial activity

Ethyl acetate extract of culture broth of *E*. *variecolor*CLB38was analyzed by disc diffusion assay to assess anti-infective potential. Strain CLB38 exhibited strong antimicrobial activity, whereas significant activity was observed against *Bacillus subtilis* (26.00±0.00 mm), *Staphylococcus aureus* (24.00±0.00 mm), *Escherichia coli* (23.33±0.33 mm) Methicillin resistant *Staphylococcus aureus* (22.33±0.33) and antifungal activity against *Candida albicans* (19.00±0.00mm). The capacity of *E*. *variecolor* CLB38 to inhibit both bacterial and fungal pathogens implies that secondary metabolites produced have a broad range of antimicrobial activity. Diameters of zone of inhibition against test pathogens are given in Table 1. The results obtained were validated with standard antibiotics, gentamicin and nystatin for antibacterial and antifungal activity, respectively. This endosymbiont might involve in defending the host against invading various pathogens and this could have extended the ability of endosymbiont to biosynthesize some chemical entities [2]. *E*. *variecolor* has been reported to exhibit bioactivities *invitro* [16,17]. Endophytic fungi inhabiting medicinal plants from Western Ghats of India were recorded to show antimicrobial and cytotoxic activities [53,54]. Similarly, endophytic actinomycetes and endophytic fungi inhabiting *C*. *latifolium* Blume which collected from the Western Ghats of India, were also reported to show antimicrobial activity in our previous studies [55,56]. Hence, this study expands our knowledge of potent hidden endosymbionts from underexplored area are a potential source of novel antimicrobial agents.

**Table 1:** Determination of antimicrobial activity of ethyl acetate fraction of endosymbiotic *Emericella variecolor* CLB38 (100 μg/disc) against test microorganisms.

Test Pathogens	Ethyl acetate extract 100 μg/disc	Gentamicin 10 μg/disc	Nystatin 100 μg/disc
***Aspergillus fumigatus* (MTCC 1811)**	17.00±0.57^f^	ND	20.66±0.57^f^
***Bacillus subtilis* (MTCC 121)**	26.00±0.00^a^	31.00±0.00^b^	ND
***Candida albicans* (MTCC 183)**	19.00±0.00^e^	ND	21.00±0.57^f^
***Escherichia coli* (MTCC 7410)**	23.33±0.33^bc^	29.33±0.33^bc^	ND
***Klebsiella pneumonia* (MTCC 7407)**	19.00±0.00^e^	27.00±0.00^de^	ND
***Listeria monocytogenes* (MTCC 839)**	23.33±0.33^c^	28.00±0.57^cd^	ND
**Methicillin resistant *Staphylococcus aureus* (ATCC 33915)**	22.33±0.33^cd^	28.66±0.33^cd^	ND
***Pseudomonas aeruginosa* (MTCC 7903)**	22.33±0.33^bcd^	25.33±0.33^e^	ND
***Salmonella typhi* (MTCC 733)**	21.00±0.00^d^	28.66±0.33^cd^	ND
***Staphylococcus aureus* (MTCC 7443)**	24.00±0.00^b^	33.00±0.00^a^	ND

Note: Values represents diameter of zone of inhibition in mm. Data represented are means from three replicates ± SE and those representing similar superscripts within columns are significantly different (ONE WAY ANOVA and Tukey’s HSD at p<0.05). ND–not determined

### Thin layer chromatography-bioautography

**Fig 6:**
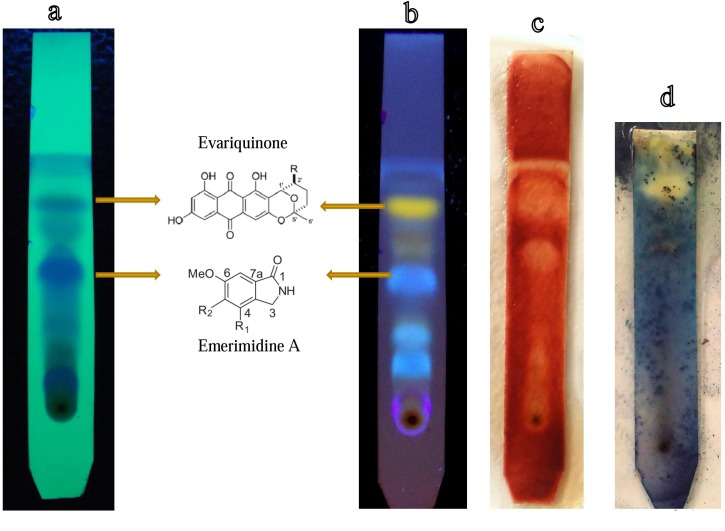
**(a, b)** Thin layer chromatogram of ethyl acetate extract of *E*. *variecolor* CLB38 at 254 nm and 364 nm, respectively. **(c)** TLC-bioautography assay of ethyl acetate extract showing zone of inhibition against Methicillin resistant *Staphylococcus aureus*. **(d)** TLC-bioautography assay of purified compound evariquinone showing zone of inhibition against *Candida albicans*.

TLC metabolic profiling of ethyl acetate extract of CLB38 displayed the presence of two intense bands under 254 nm and 365 nm. Band at *R*_*f*_ 0.58 and *R*_*f*_ 0.46 appeared yellow and blue in color, respectively (Fig 6). These intense bands might be due to the increased production of bioactive metabolites. During TLC-bioautography assay, these two bands exhibited clear zone of inhibition where the medium was pre-inoculated with MRSA and TTC agent. This confirms antimicrobial compounds present in the ethyl acetate fraction which was active against MRSA. Antimicrobial compounds detection by TLC-bioautography method is one of the economical, simplest and reproducible method for biodiscovery from nature products [57,58].Antimicrobial TLC bioautography was used as bio-assay to monitor antimicrobial compound purification process by column and thin layer chromatography. Biodiscovery of antimicrobial producing fungal endosymbionts associated with *C*. *latifolium* Blume is valuable for industrial interest and for basic research.

### Purification of antimicrobial compounds

Purification of antimicrobial compounds was carried out in two step purification process. Thirteen fractions (F1~F13) derived from silica gel column chromatography were analyzed by TLC–bioautography for antimicrobial potential. Fractions F3~F5and F8~F10 showed similar TLC patterns differ from each group which are active against MRSA. Since MRSA is a multidrug-resistant pathogen, we used as an indicator organism to assess the anti-infective potential of these compounds. Thus, these two groups of fractions were combined and re-chromatographed over silica gel column separately in order to obtain highly purified compounds. Further, from the secondary step of purification, the eluted fractions from each group were combined, evaporated to dryness which furnished two compounds as yellow crystals (group A fractions; compound (1) and colorless crystals (group B fractions; compound 2).

### Structure elucidation of antimicrobial compounds

**Fig 7:**
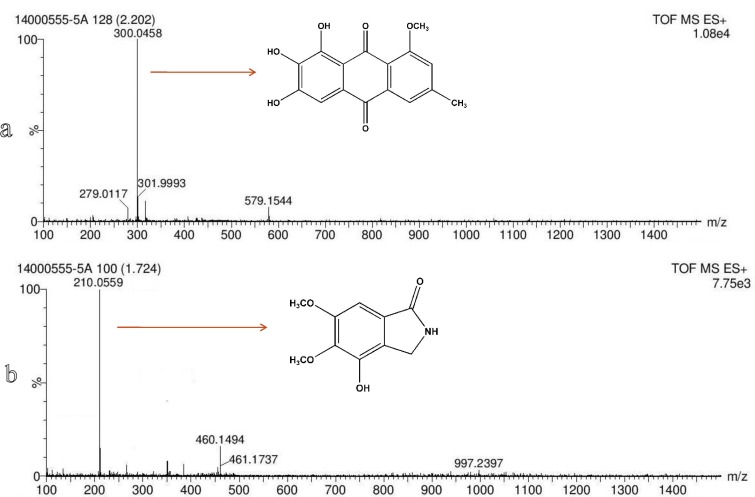
**ESI-TOF-MS of (1) Evariquinone and (2) Emerimidine A showing major molecular ion peaks**.

**Fig 8:**
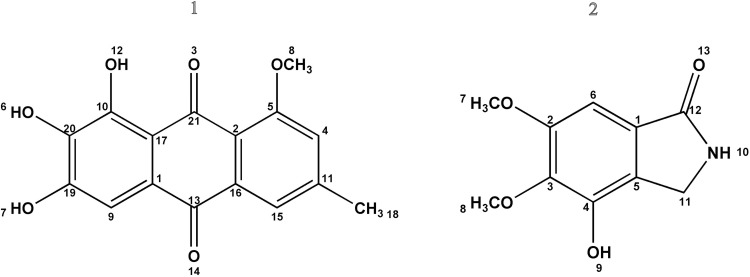
**Structure of (1) Evariquinone and (2) Emerimidine A**.

Evariquinone (1) was isolated as pale yellow amorphous powder. Its molecular formula was deduced to be C_16_H_12_O_6_ by *m/z* 300.0458 (Fig 7). ^1^H NMR chemical shifts (DMSOd_6_, 400 MHz, δ ppm): δ 2.421 (3H, s,CH_3_), 3.97 (3H, s, OCH_3_), 6.951 (1H, s, Ar-H), 7.212 (1H, d, Ar-H), 7.564 (1H, s, Ar-H), 12.513 (2H, s, Ar-H) 12.766 (1H, s, Ar-H) (S1 Fig) indicates the presence of a core evariquinone structure that was supported by a literature precedent (Fig 8) [17,59]. Emerimidine A (2) was isolated as pale colorless amorphous powder. Its molecular formula was deduced to be C_10_H_12_NO_4_ by *m/z*210.0559 [M+H]^+^(Fig 7).^1^H NMR chemical shifts (DMSO d_6_, 400 MHz, δ ppm):3.772 (3H, s, OCH_3_), 3.796 (3H, s, OCH_3_), 4.147 (2H, s, CH_2_), 6.989 (1H, s, Ar-H), 8.267 (1H, s, NH), 11.989 (1H,s, OH)(S1 Fig) indicates the presence of a core emerimidine structure that was supported by a literature precedent (Fig 8) [60].The purified metabolites significantly inhibited MRSA, *S*. *aureus*, *L*. *monocytogenes*, *Pseudomonas aeruginosa*, *Staphylococcus epidermidis* and *Candida albicans* with MIC values of 3.15 to 12.5 μg/ml. MIC values of the purified compounds against test microorganisms are given in Table 2 and Table 3.

**Table 2:** Minimum inhibitory concentration (MIC in μg/ml) of purified compound Evariquinone from endosymbiotic *E*. *variecolor* CLB38 against test microorganisms.

**Test Pathogens**	**Evariquinone (C**_**10**_**H**_**11**_**NO**_**4**_**)**
***Aspergillus fumigatus* (MTCC 1811)**	12.5
***Bacillus subtilis* (MTCC 121)**	3.12
***Candida albicans* (MTCC 183)**	3.12
***Klebsiella pneumonia* (MTCC 7407)**	12.5
**Methicillin resistant *Staphylococcus aureus* (ATCC 33915)**	3.12
***Pseudomonas aeruginosa* (MTCC 7903)**	12.5
***Salmonella typhi* (MTCC 733)**	6.25

**Table 3:** Minimum inhibitory concentration (MIC in μg/ml) of purified compound Emerimidine A from endosymbiotic *E*. *variecolor* CLB38 against test microorganisms.

**Test Pathogens**	**Emerimidine A (C**_**16**_**H**_**12**_**O**_**6**_**)**
***Aspergillus fumigatus* (MTCC 1811)**	12.5
***Bacillus subtilis* (MTCC 121)**	6.25
***Candida albicans* (MTCC 183)**	6.25
***Klebsiella pneumonia* (MTCC 7407)**	12.5
**Methicillin resistant *Staphylococcus aureus* (ATCC 33915)**	6.25
***Pseudomonas aeruginosa* (MTCC 7903)**	12.5
***Salmonella typhi* (MTCC 733)**	6.25

ESI-TOF-MS analysis of compound **1** exhibited a molecular ion peak *m/z* 300.0458 corresponding to the mass of evariquinone [17] whereas compound **2** exhibited a molecular ion peak *m/z* 210.0559 corresponding to the mass of emerimidine A. Literature survey of TOF-MS spectral data with that of emerimidine A indicates that, the metabolite could be a derivative of isoindolones [56]. TOF-MS is a powerful tool for accurate and rapid identification of known compounds, i.e. dereplication which has a great importance towards discovery of new antimicrobial agents [61–63]. This strategy enables to minimize wasting attempt on isolation of known bioactive compounds. To our best knowledge, this work is the first report on systematic analyses of endosymbiotic *E*. *variecolor* CLB38 to explore its antimicrobial potential via the implication of PKS type I gene and chromatographic strategy to yield two polyketide antimicrobial metabolites. Development of these compounds as antimicrobial drugs after essential evaluation like preclinical trials and toxicity may enlights new ideas to combat multidrug-resistant pathogens.

Additionally, evariquinone is well known to possess anti-proliferative activity. It has been reported from marine sponge derived *Emericella variecolor* [17] and endophytic *Aspergillus versicolor* with a wide range of bioactivity [50]. Indeed, isoindolones derivatives also possess biological activities. Specifically, a series of emerimidine (A-B) derived from endophytic fungus *Emericella* sp. HK-ZJ which isolated from the inner bark of the mangrove plant *Aegiceras corniculatum* (Myrsinaceae) has been reported to show anti-influenza A viral (H_1_N_1_) activity [51]. Thus, from the present study, it suggests that the antimicrobial potential of these isolated metabolites might play a significant role in symbiotic benefits of endosymbiont to the host. Besides the discovery of natural products from the genus *Emericella*, the ability of *E*. *variecolor* CLB38 to afford reported compounds which additionally support the evidence that, fungal endosymbionts are entailed in the biosynthesis of antimicrobial agents. The chemical components bear in this host might impact endosymbionts to produce potential antimicrobial compounds. The need for future biological studies, like cytotoxicity, anti-malarial, antioxidant and chemical profiling of secondary metabolites of *E*. *variecolor* CLB38 could be inspired towards drug development. Clearly, formulation and development of new technologies are needed for employing them in agricultural and pharmaceutical fields. Extensive exploration of various nature antimicrobial compounds from endosymbiotic fungi associated with medicinal plants of the Western Ghats might provide high insight to the evolution of mutualism and endophyte-plant interactions. Therefore, the present study expands our knowledge of potential hidden endosymbiotic fungi from underexplored area are a promising source of novel antimicrobial drugs.

## Conclusion

This work demonstrates a holistic strategy for the rapid detection of antimicrobial polyketide metabolites from endosymbiotic fungi. *E*. *variecolor* CLB38 is an anticipated source of antimicrobial polyketide agents which can be exploited in medical and industrial applications. PKS type I gene is a functional gene of *E*. *variecolor* CLB38 which performs a significant role in fungal endophytic secondary metabolite biosynthesis. Exploiting PKS biosynthetic genes to discover novel anti-infective agents are productive and fascinating in drug development area. This work also highlights the importance of ITS2 RNA secondary structure modeling as a potential molecular marker which helps to distinguish fungal endosymbionts with other pathogenic or free-living forms.

## Supporting information

S1 Fig **(1) ^1^H NMR spectrum of Evariquinone, (2) ^1^H NMR spectrum of Emerimidine A**.
(TIFF)
