# Supplementary figures and images for: Chemogenomics driven discovery of endogenous polyketide anti-infective compounds from endosymbiotic Emericella variecolor CLB38 and their RNA secondary structure analysis
Source: PLoS One. 2017 Feb 28;12(2):e0172848. doi: 10.1371/journal.pone.0172848 (PMC5330499; doi:10.1371/journal.pone.0172848)

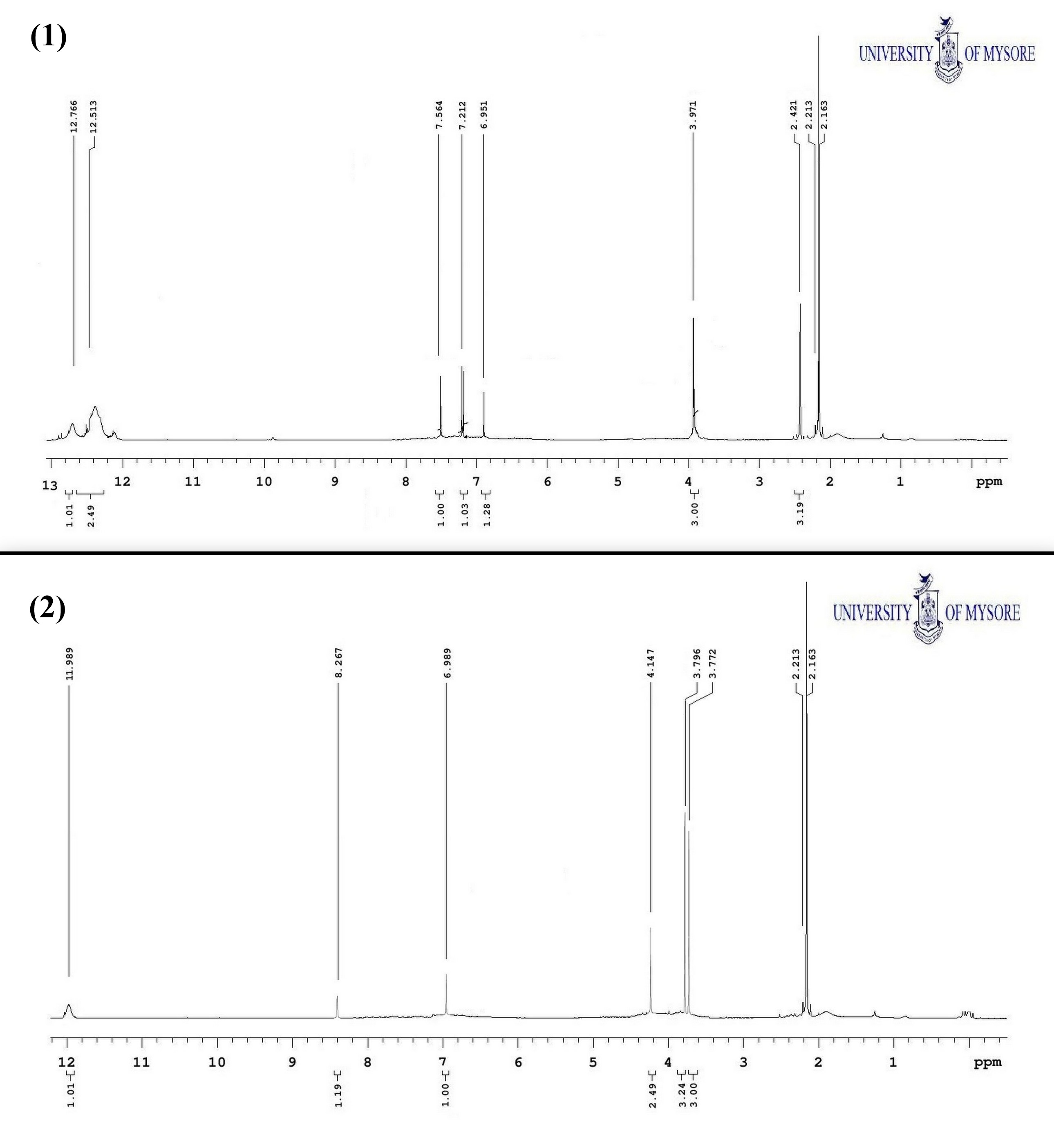

Supplement: S1 Fig — (1) 1H NMR spectrum of Evariquinone, (2) 1H NMR spectrum of Emerimidine A. (TIFF) [file pone.0172848.s001.tiff]
